# Supplementary material for: SyNDI: synchronous network data integration framework
Source: BMC Bioinformatics. 2018 Nov 6;19:403. doi: 10.1186/s12859-018-2426-5 (PMC6219086; doi:10.1186/s12859-018-2426-5)
Supplement: Supplementary file 5 — Files of the example presented in Additional file 4. This additional file is a zip package that contains all files used in Additional file 4. Detailed descriptions of each of these files are presented in a README file included in this zip package. (GZ 30411 kb) [file 12859_2018_2426_MOESM5_ESM.gz › SyNDITools/Meme2Fimo/meme_4.9.0_4.tar.gz!/meme_4.9.0/doc/iupac.html]

MAST IUPAC Ambiguous Characters


# MAST IUPAC Ambiguous Characters

| optional  letter | matches | |
| --- | --- | --- |
|  | DNA | protein |
| B | CGT | DN |
| D | AGT |  |
| H | ACT |  |
| K | GT |  |
| M | AC |  |
| N | ACGT |  |
| R | AG |  |
| S | CG |  |
| U | T | ACDEFGHIKLMNPQRSTVWY |
| V | CAG |  |
| W | AT |  |
| X |  | ACDEFGHIKLMNPQRSTVWY |
| Y | CT |  |
| Z |  | EQ |
| \* | ACGT | ACDEFGHIKLMNPQRSTVWY |
| - | ACGT | ACDEFGHIKLMNPQRSTVWY |
